# The Makers of Dentistry

**Published:** 1901-11

**Authors:** 


"THE MAKERS OF DENTISTRY." By Charles McManus, D.D.S., Hartford, Conn.—A paper entitled "The Makers of Dentistry," was read by Charles McManus, D.D.S., before the Hartford Dental Society, Tuesday evening, September 3, dealing with the history of dentistry and illustrated with *lantern-slide portraits of over seventy-five* celebrated American and European dentists. Among the number were A. Paré, Pierre Fauchard, John Hunter, Joseph Lemaire, James Gardette, John Greenwood, Paul Revere, John Randall, Leonard Koecker, Horace H. Hayden, Chapin A. Harris, Horace Wells, John M. Riggs, G. Q. Colton, Sir John Tomes, Sir Edwin Saunders, Thomas W. Evans, Elisha Townsend, S. P. Cutler, Edward Maynard, Jas. Taylor, Amos Westcott, Joseph Richardson, E. J. Dunning, Thos. B. Gunning, Asa Hill, R. W. Varney, S. C. Barnum, Marshall H. Webb, J. B. Morrison, B. J. Bing, W. H. Dwinelle, W. H. Atkinson, W. W. Allport, W. G. A. Bonwill, J. H. McQuillen, Thomas W. Parsons, S. S. White, J. E. Garretson, W. Herbst, W. H. Morgan, H. J. McKellops, Frank Abbott, Cushing, Emile Magitot, Charles S. Tomes, W. D. Miller, and many other well-known dentists who have contributed to the making of the profession.

